# Cholesteatoma-associated fibroblasts modulate epithelial growth and differentiation through KGF/FGF7 secretion

**DOI:** 10.1007/s00418-012-0947-y

**Published:** 2012-04-06

**Authors:** Salvatore Raffa, Laura Leone, Cristina Scrofani, Simonetta Monini, Maria Rosaria Torrisi, Maurizio Barbara

**Affiliations:** 1 Istituto Pasteur-Fondazione Cenci Bolognetti, Department of Clinical and Molecular Medicine, Sapienza University of Roma, Piazza Sassari 3, 00161 Rome, Italy; 2 Department of Neuroscience, Mental Health and Sensory Organs, Sapienza University of Rome, Rome, Italy; 3 Azienda Ospedaliera S. Andrea, Rome, Italy

**Keywords:** Keratinocyte growth factor, Fibroblast growth factor, Cholesteatoma, Cultured human fibroblasts, Metalloproteases

## Abstract

The keratinocyte growth factor (KGF/FGF7), produced by stromal cells, is a key paracrine mediator of epithelial proliferation, differentiation and migration. Expression of the growth factor is increased in wound healing and in hyperproliferative epithelial diseases, as a consequence of the activation of dermal fibroblasts by the inflammatory microenvironment. The middle ear cholesteatoma, an aural epidermal pathology characterized by keratinocyte hyperproliferation and chronic inflammation, may represent a model condition to study the epithelial-mesenchymal interactions. To develop an in vitro model for this disease, we isolated and characterized human primary cultures of fibroblasts associated with the cholesteatoma lesion, analyzing their secretory behaviour and degree of differentiation or activation. Compared to the perilesional or control normal fibroblasts, all cultures derived from cholesteatoma tissues were less proliferating and more differentiated and their highly variable activated phenotype correlated with the secretion of KGF as well as of metalloproteases 2 and 9. Culture supernatants collected from the cholesteatoma-associated fibroblasts were able to increase the proliferation and differentiation of human keratinocytes assessed by the expression of Ki67 and keratin-1 markers. The single crucial contribution of the KGF released by fibroblasts on the keratinocyte biological response was shown by the specific, although partial, block induced by inhibiting the KGF receptor or by immunoneutralizing the growth factor. Altogether, these results suggest that the activation of the stromal fibroblasts present in the pathological tissue, and the consequent increased secretion of KGF, play a crucial role in the deregulation of the epidermal proliferation and differentiation.

## Introduction

The keratinocyte growth factor (KGF/FGF7) is a member of the fibroblast growth factor family and a major paracrine mediator of the epidermal homeostasis through regulation of the keratinocyte proliferation, differentiation and migration (Finch et al. 1989; Marchese et al*.* 1990; Ceccarelli et al. 2007). Produced by stromal cells, KGF binds exclusively to the KGFR, a splicing transcript variant of the fibroblast growth factor receptor 2 (FGFR2b) (Miki et al*.* 1992). In addition to its physiologic role, KGF is up-modulated in its expression in human wound healing during the process of re-epithelization (Marchese et al. 1995). In previous papers from our group, we have reported that KGF expression is also increased in hyperproliferative epithelial diseases, such as psoriasis and acanthoma, as a consequence of the activation of dermal fibroblasts by the inflammatory microenvironment (Kovacs et al. 2005, 2006). These results are consistent with the current knowledge that, in different epithelial pathological conditions and in tumorigenesis, the activation of the stromal cells is able to sustain the keratinocyte hyperproliferation and altered differentiation through the secretion of soluble paracrine growth factors and cytokines (Räsänen and Vaheri 2010)*.*

Among the hyperproliferative epithelial diseases, the middle ear cholesteatoma (CHO) is an aural lesion, which may grow and invade the surrounding stroma, including bone and middle/inner ear structures (Olszewska et al. 2004). Although different studies have shown the invasive and hyperproliferative behaviour of the CHO epidermal tissue (Bujia et al. 1993; Vennix et al. 1996), very little is still known about the molecular mechanisms involved in the pathogenesis of the disease. Others and we have suggested that the proliferation and migration of the keratinocytes in CHO might be mediated by an altered release of autocrine and paracrine growth factors and a modulated expression of their receptors (Kojima et al. 1994; Tanaka et al. 1999; Yetiser et al. 2002). Among them, KGF and KGFR expression appear up-modulated in CHO tissue (Kojima et al. 1996; Ishibashi et al. 1997; Yamamoto-Fukuda et al*.* 2003, 2010; Barbara et al. 2008; d’Alessandro et al. 2010) and the efficacy of a common therapeutic treatment of the lesion is related to a down-modulation of the growth factor (Yamamoto-Fukuda et al*.* 2008). Therefore, it is possible that the activation of the stromal fibroblasts associated to the lesion and the consequent release of KGF could play a role in the deregulation of the epidermal proliferation and differentiation, which characterizes the CHO tissue.

The development of in vitro models of epithelial-mesenchymal interaction may greatly contribute to elucidate the effects mediated by the stromal fibroblasts on the epithelial cells and the molecular mechanisms involved in the CHO pathogenesis. To this aim, in this study, we established and characterized human primary cultures of CHO-associated fibroblasts analyzing their growth and secretory behaviour and their degree of differentiation/activation. In addition, we evaluated the ability of their culture supernatants to modulate the biological response of human keratinocytes, demonstrating the key contribution of the KGF secreted in the medium in modulating this response. Our results indicate that CHO-associated fibroblasts might be able to sustain the growth and invasive behaviour of the lesion through the secretion of paracrine diffusible factors, such as KGF, and of pro-invasive molecules, such as metalloproteases.

## Materials and methods

### Patients and tissue samples

Tissue samples were obtained from middle ear cholesteatoma specimens (CHO-AFs) collected during surgical procedures performed on 11 consecutive patients. The control samples were obtained from skin of the medial external auditory canal (MEAC-Fs) during surgical procedure performed on a patient with otosclerosis and from non-auricolar skin (NAS-Fs) by punch-biopsy performed on a patient with abdominal aortic aneurysm. The clinical and demographic characteristics of the enrolled patients and site of sampling are described in Table 1. All patients were extensively informed and gave written consent for the investigations. The study was approved by the ethics committee of the University Hospital where it was conducted. Tissue samples were examined by toluidine blue semithin sections and assessed independently by two pathologists.

**Table 1:** Clinical and demographic features of the enrolled patients, site and characteristics of tissue sampling and results of the analysis

Specimen code	Age (yrs)	Sex	Clinical features	Extent of dermal inflammatory infiltrate (score)^a^	Site of sampling	L:E index	G2/M phase (%)	MMP-2 ng/1 x 10^6^ cells (cellular homogenate)	MMP-9 ng/1 x 10^6^ cells (cellular homogenate)	α-SMA expression (%)	KGF pg/1 x 10^6^ cells (culture supernatants)
#1410	52	F	Cholesteatoma; second revision surgery	2	Petrosectomy cavity	0.920	1.44	25.6	103.35	93.3	86.8
#2110	13	M	Cholesteatoma; first revision surgery	1	Hypotimpanum	0.462	4.52	10.2	38.72	10.6	68.87
#0926	47	F	Cholesteatoma; second revision surgery	2	Mastoidectomy cavity	0.248	1.35	5.0	15.99	8.7	15.99
#2810	63	F	Cholesteatoma; first revision surgery	2	Mastoidectomy cavity, epitympanum, mesotympanum	0.219	5.71	13.0	41.45	6.4	83.9
#1811	16	F	Cholesteatoma; first surgery	1	Epitympanum, mesotympanum	0.214	8.72	11.6	32.47	14.4	34.8
#2011	16	M	Cholesteatoma; first revision surgery	1	Antrum, epitympanum	0.213	4.6	5.6	11.11	10.9	34.55
#0311	47	F	Cholesteatoma; first revision surgery	1	Antrum	0.167	5.21	6.0	16.76	5.5	81.82
#2010	32	M	Cholesteatoma; first revision surgery	1	Mastoidectomy cavity	0.101	6.92	5.3	16.64	4.6	40.98
#1211	72	F	Cholesteatoma; first surgery	0	Mastoid mesotympanum	0.075	9.03	3.7	11.98	0.5	18.99
#0811	39	M	Cholesteatoma; first surgery	2	Mastoid, antrum, epitympanum, mesotympanum	0.212	6.94	8.6	44.84	24.1	93.11
#0811p				0	Perilesional skin of #0811	0.109	18.4	5.5	22.5	0.8	18.99
#1011	27	F	Cholesteatoma; first surgery	1	Mastoid, antrum, epitympanum, mesotympanum	0.131	2.04	4.8	11.94	11.9	66.19
#1011p				0	Perilesional skin of #1011	0.181	9.36	2.5	10.1	0.2	14
NAS-Fs	25	M	Abdominal aortic aneurysm	0	Normal skin of back	0.149	12	4.1	15.2	0.9	24.54
MEAC-Fs	70	F	Otosclerosis	0	Normal skin of medial external auditory canal	0.086	12.7	2.5	8.14	1.6	20.01

^a^d’Alessandro et al. (2010)

### Cell cultures and treatments

Primary cultures of human fibroblasts were obtained from middle ear cholesteatoma specimens (CHO-AFs). Control cultures were derived from perilesional tissue of cholesteatomatous patients (perilesional-Fs), from normal human skin of the medial external auditory canal (MEAC-Fs) and from non-auricolar skin (NAS-Fs).

Tissue samples were cut into small pieces, digested with dispase 0.1 mg/ml and collagenase I 0.35 % for 45 min at 37 °C, pelleted, resuspended and maintained in Dulbecco’s modified Eagle medium (DMEM) containing 10 % fetal bovine serum (FBS) and antibiotics. Primary cultures were obtained and propagated up to the second passage.

To induce fibroblast differentiation, the cultures of CHO-AFs were maintained in DMEM without serum for 48 h in the presence of TGFβ 50 ng/ml (PeproTech Inc., Rocky Hill, NY, USA).

Supernatants (SNs) obtained from primary cultured CHO-AFs, MEAC-Fs, NAS-Fs and perilesional-Fs kept in serum-free medium for 48 h were collected and frozen at −80 °C until use. SNs were also obtained from two primary cultured CHO-AFs kept in serum-free medium in presence of TGFβ 50 ng/ml for 48 h.

The human keratinocyte cell line HaCaT was cultured in DMEM supplemented with 10 % FBS and antibiotics. Primary cultures of normal human keratinocytes (NHKs) were derived from skin biopsies as described previously (Belleudi et al. 2011) and maintained in Medium 154-CF (Cascade Biologics, Portland, OR, USA) supplemented with Human Keratinocyte Growth Supplement (HKGS, Cascade Biologics) plus antibiotics and Ca^2+^ 0,03 mM (CascadeBiologics Inc.).

For proliferation assay, HaCaT cells were treated for 48 h at 37 °C with 20 ng/ml recombinant KGF (Upstate Biotechnology, Lake Placid, NY, USA) or SNs collected from cultured CHO-AFs, perilesional-Fs, MEAC-Fs and NAS-Fs as above. For evaluation of the cellular differentiation, confluent HaCaT cells were treated for 48 h at 37 °C with 20 ng/ml KGF or the SNs collected as above.

For inhibition of KGFR activity, HaCaT cells were pre-incubated with a specific FGFR tyrosine kinase inhibitor SU5402 25 μM (Calbiochem, Nottingham, UK) for 1 h before treatments with KGF or SNs in presence of SU5402. To selectively block the KGF activity, we used a neutralizing rabbit polyclonal antibodies to KGF (C-19, Santa Cruz Biotechnology, Santa Cruz, CA, USA) (10 μg/ml in culture medium).

### Immunofluorescence

Cells grown on coverslips were fixed with 4 % paraformaldehyde followed by treatment with 0.1 M glycine for 20 min at 25 °C and with 0.1 % Triton X-100 for additional 5 min at 25 °C to allow permeabilization. Cells were then incubated with the following primary antibodies: anti-vimentin (1:50 in PBS; Dako, Glostrup, Denmark), anti-cytokeratins (1:50 in PBS; Dako), anti α-SMA (1:50 in PBS; Dako) monoclonal antibodies, anti-Ki67 (1:50 in PBS; Zymed Laboratories, Inc, San Francisco, CA) and anti-keratin 1 (1:50 in PBS; Covance, Emeryville, CA, USA) rabbit polyclonal antibodies.

The primary antibodies were visualized, after appropriate washing with PBS, by using goat anti-mouse IgG–FITC (1:50 in PBS; Cappel Research Products, Durham, NC, USA), goat anti-rabbit IgG–Texas Red (1:200 in PBS; Jackson Immunoresearch Laboratories, West Grove, PA, USA) and goat anti-rabbit IgG–FITC (1:500 in PBS; Cappel Research Products), for 30 min at 25 °C. Nuclei were stained with 4′-6′-diamido-2-phenylindole dihydrochloride (DAPI) (1:10,000 in PBS, Sigma Chemical Co., St. Louis, MO, USA). Coverslips were finally mounted with Mowiol in PBS for observation.

Fluorescence signals were analyzed by conventional fluorescence or by scanning cells in series of 0.5 μm sequential sections with an ApoTome System (Zeiss, Oberkochen, Germany) connected with an Axiovert 200 inverted microscope (Zeiss); image analysis was then performed by the Axiovision software (Zeiss).

The percentage of Ki67- or K1- positive cells was analyzed counting for each treatment a total of 500 cells, observed in ten microscopic fields randomly taken from three different experiments. Results have been expressed as mean values ± SE.

### Western blot analysis

Cells were lysed in a buffer containing 50 mM HEPES pH 7.5, 150 mM NaCl, 1 % glycerol, 1 % Triton X-100, 1.5 mM MgCl_2_, 5 mM EGTA, supplemented with protease inhibitors (10 mg/ml aprotinin, 1 mM PMSF, 10 mg/ml leupeptin), and phosphatase inhibitors (1 mM sodium orthovanadate, 20 mM sodium pyrophosphate, 0.5 M NaF); 50 μg of total protein were resolved under reducing conditions by 8 % SDS-PAGE and transferred to reinforced nitrocellulose (BA-S 83, Schleider and Schuell, Keene, NH, USA). The membranes were blocked with 5 % non-fat dry milk in PBS 0.1 % Tween 20, and incubated with anti-KGF (H-73, Santa Cruz) polyclonal antibodies, followed by enhanced chemiluminescence detection (ECL; Amersham, Arlington Heights, IL). The membranes were rehydrated by being washed in PBS-Tween 20, stripped with 100 mM mercaptoethanol and 2 % SDS for 30 min at 55 °C, and probed again with anti-actin (Sigma) monoclonal antibody, to estimate the protein equal loading.

Densitometric analysis was performed using Quantity One Program (Bio-Rad Headquarters, Hercules, CA, USA). Briefly, the signal intensity for each band was calculated and the background subtracted from experimental values. The resulting values were then normalized and expressed as fold increase with respect to the control and mean values ± SE from three different experiments in triplicate.

### Primers

Oligonucleotide primers for target genes and for the housekeeping gene were chosen with the assistance of the Oligo 5.0 computer program (National Biosciences, Plymouth, MN) and purchased from Invitrogen. The following primers were used: for *KGF* target gene: 5′-CACCAGGCAGACAACAGACAT-3 (sense), 5′-GTAAGTTCAGTTGCTGTGACGCT-3′ (anti-sense). For each primer pair, we performed no-template control and no-reverse-transcriptase control (RT negative) assays, which produced negligible signals.

### RNA extraction and cDNA synthesis

RNA was extracted using the TRIzol method (Invitrogen, Carlsbad, CA) according to manufacturer’s instructions and eluted with 0.1 % diethylpyrocarbonate (DEPC)-treated water. Total RNA concentration was quantitated by spectrophotometry and the quality was assessed by measuring the optical density ratio at 260/280 nm. RNA samples were stored at −80 °C. After denaturation in DEPC-treated water at 70 °C for 10 min, 1 μg of total RNA was used to reverse transcription using iScript™ cDNA synthesis kit (Bio-Rad) according to manufacturer’s instructions.

### PCR amplification and real-time quantitation

Real-time PCR was performed using the iCycler Real-Time Detection System (iQ5 Bio-Rad) with optimized PCR conditions. The reaction was carried out in 96-well plate using iQ SYBR Green Supermix (Bio-Rad) adding forward and reverse primers for each gene and 1 μl of diluted template cDNA to a final reaction volume of 15 μl. All assays included a negative control and were replicated three times. The thermal cycling programme was performed as follows: an initial denaturation step at 95 °C for 3 min, followed by 45 cycles at 95 °C for 10 s and 60 °C for 30 s. Real-time quantitation was performed with the help of the iCycler IQ optical system software version 3.0a (Bio-Rad), according to the manufacturer’s manual. The relative expression of the housekeeping gene was used for standardizing the reaction. The comparative threshold cycle (*C*_t_) method was applied to calculate the fold changes of expression compared to control cells. Results are reported as mean ± standard deviation (SD) from three different experiments in triplicate.

### Flow cytometry for cell cycle

For each culture, the fibroblasts were plated sparsely, trypsinized, pelleted and resuspended in 70 % ethanol in PBS and stored at 4 °C overnight. Cells were washed with PBS, resuspended in propidium iodide (PI) staining solution (50 μg/ml) and RNAse A (100 Kunitz/ml) (Miltenyi Biotec GmbH, Bergisch Gladbach, Germany) and incubated in the dark for 40 min at room temperature. At least 20,000 cells for each culture were collected and analyzed with MACSQuant^®^ Analyzer flow cytometer (Miltenyi Biotec GmbH). Cell cycle distribution was calculated with MACSQuantify^®^ software (Miltenyi Biotec GmbH).

### Morphological analysis

For the fibroblast morphological analysis, three differentiation stages were evaluated: FI = small spindle shaped cell, FII = small epithelioid cells, FIII = large epithelioid cells (Bayreuther et al. 1988). The L:E differentiation index was calculated analyzing the fibroblasts in early (E: FI + FII) and late (L: FII/FIII + FIII) differentiation state (Herskind and Rodemann 2000). The culture samples were observed on a Zeiss Axiovert 200 inverted microscope equipped with differential interference contrast (DIC) optics. Quantitative analysis was performed by counting, for each cell culture, a total of at least 250 cells observed in five microscopic fields randomly taken from three different experiments.

### ELISA

Matrix metalloproteinases-2 (MMP-2) and -9 (MMP-9) in cellular homogenates from CHO-AFs, MEAC-Fs, NAS-Fs and perilesional-Fs were quantified using the human MMP-2 ELISA kit and human MMP-9 ELISA kit (Quantikine^®^; R&D Systems, Minneapolis, MN) according to the manufacturer’s protocol. Standard curves for each ELISA experiment were prepared from duplicate wells with increasing concentrations of MMP-2 (0–0.78–1.56–3.13–6.25–12.5–25–50 ng/ml), or MMP-9 (0–0.312–0.625–1.25–2.5–5–10 ng/ml). The results were normalized for the number of cells contained in each sample and were expressed as nanograms/1 × 10^6^ cells. KGF in the SNs collected from the cultures was quantified using the human KGF ELISA kit (Quantikine^®^; R&D Systems, Minneapolis, MN) according to the manufacturer’s protocol. A standard curve for each ELISA experiment was prepared from duplicate wells with increasing concentrations of KGF (0–31.2–62.5–125–250–500–1,000–2,000 pg/ml), using the protocol described above. The results were normalized for the number of cells contained in each sample and were expressed as picograms/1 × 10^6^ cells. Each sample was analyzed in triplicate. Mann–Whitney test was performed to evaluate significant differences between samples. *P* < 0.05 was considered statistically significant.

### Statistical methods

Mann–Whitney non-parametric test was used to compare variables that do not assume Gaussian distribution. Student’s *t* test was used to compare variables that assume Gaussian distribution. All the correlation measures were evaluated by the Pearson test (*r*) and by linear analysis of regression curve. *P* values < 0.05 were assumed as statistically significant.

## Results

### Establishment and characterization of primary cultures of fibroblasts derived from cholesteatoma lesions and assessment of their differentiated phenotype

To analyze the behaviour of the stromal fibroblasts associated with the cholesteatoma (CHO) hyperproliferative epidermal disease, we isolated and propagated in culture primary fibroblasts obtained from middle ear CHO specimens (CHO-AFs). For control samples, we established fibroblast cultures from the perilesional tissue of cholesteatomatous patients (perilesional-Fs) as well as from normal human skin of the medial external auditory canal (MEAC-Fs) and from non-auricolar skin (NAS-Fs). All the cells were isolated and cultured as described in “Materials and methods”. The number of the cultures analyzed throughout the study and the clinical and demographic characteristics of the enrolled patients and site of sampling are described in Table 1.

**Fig. 1:**
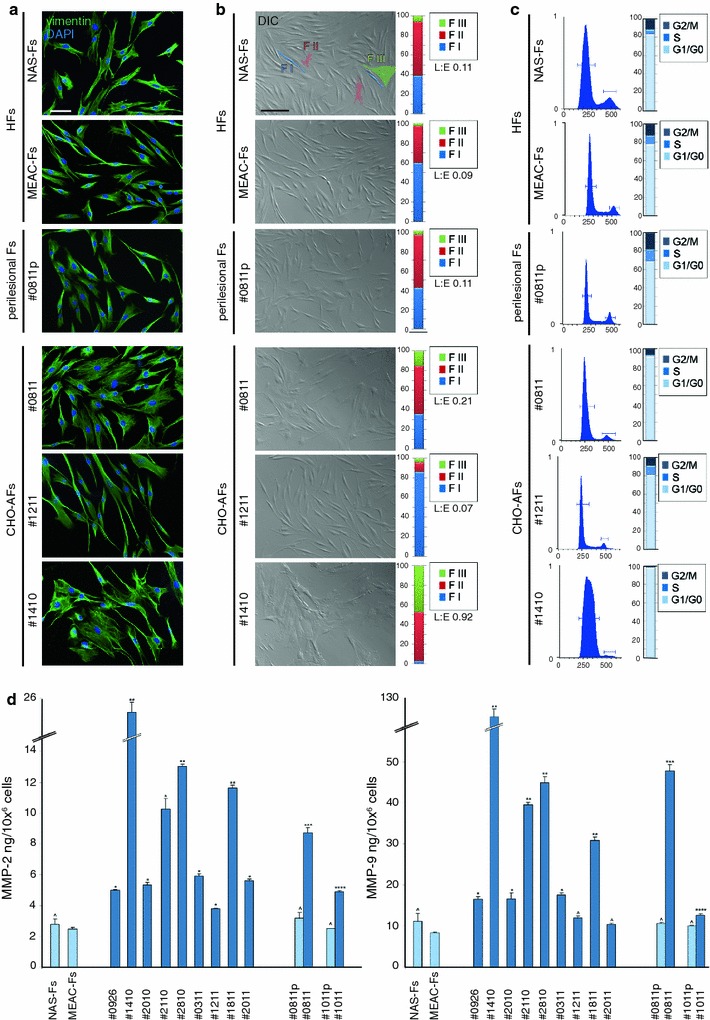
Characterization of the primary cultures of cholesteatoma-associated fibroblasts. **a** Immunofluorescence analysis of expression of the mesenchymal marker vimentin on representative example of different cultures of human fibroblasts isolated from non-auricolar skin (NAS-Fs), from normal skin of the medial external auditory canal (MEAC-Fs), from middle ear cholesteatoma samples (CHO-AFs) and from perilesional tissue of cholesteatomatous patients (perilesional-Fs). in all cultures, the cells are positive for vimentin staining, which appears as perinuclear cytoplasmic bundles of filaments. Nuclei are stained with DAPI. *Bar* 20 μm. **b** Morphologycal analysis of the primary cultures by differential interference contrast microscopy. Three differentiation stages were defined: FI, small spindle shaped cell (*blue*); FII: small epithelioid cells (*red*); FIII: large epithelioid cells (*green*). The Late:Early (L:E) ratio was calculated. The quantitative analysis indicate that among the CHO-AFs the percentages of cells in the more differentiated stages FII, FIII and the L:E ratio are highly variable. In contrast, in the control perilesional-Fs and in MEAC-Fs or NAS-Fs the percentages are homogenous. *Bar* 50 μm. **c** Fibroblast cultures were stained with PI and analyzed for cell cycle distribution with flow cytometry. The percentage of cells in G2/M results lower in CHO-AFs compared to control cultures and the more quiescent phenotype frequently corresponds to a higher L:E ratio. **d** MMP-2 and -9 quantitation by ELISA test on cellular homogenates from CHO-AFs, perilesional-Fs, MEAC-Fs, NAS-Fs. The MMP-2 and -9 levels in CHO-AFs were detected in various amounts but are higher respect to the cellular homogenates from control fibroblasts. Results reported in graph represent the mean values ± SE. Mann–Withney test was performed and significance level has been defined as described in “Materials and methods”. Statistics for MMP-2: ^*p* = NS versus MEAC-Fs and *p* = NS versus MEAC-Fs; *p < 0.05 versus MEAC-Fs; **p < 0.01 versus MEAC-Fs; ***p < 0.01 versus MEAC-Fs and *p* < 0.01 versus corresponding perilesional-Fs, ****p < 0.05 versus MEAC-s and *p* < 0.05 versus corresponding perilesional-Fs. Statistics for MMP-9: ^*p* = NS versus MEAC-Fs; *p < 0.05 versus MEAC-Fs; **p < 0.01 versus MEAC-Fs; ***p < 0.001 versus MEAC-Fs and *p* < 0.001 versus corresponding perilesional-Fs, ****p < 0.05 versus MEAC-s and *p* < 0.05 versus corresponding perilesional-Fs

The mesenchymal phenotype of the primary cultures and their purity were first confirmed by immunofluorescence assessment of the expression of vimentin, a component of the intermediate filaments widely used as a fibroblastic marker: the analysis showed that, in all cultures, cells were positive for vimentin staining, displaying a signal compatible with the structure and localization of perinuclear cytoplasmic bundles of filaments (Fig. 1a). To rule out the presence of epithelial cells in the cultures, cells were incubated with anti-cytokeratins antibodies which recognize the intermediate filaments of epithelial cells: no signal was detected for cytokeratins (not shown) confirming the mesenchymal phenotype of all the isolated primary cultures.

To analyze possible real differences in the biological characteristics of our cultures, which may reflect the in vivo behaviour, as well as to avoid in vitro differentiation, we used all cultures at the second passage (P2). To evaluate the degree of differentiation of the cells, first we utilized and adapted a morphological characterization (Bayreuther et al. 1988) proposed to distinguish in vitro different differentiation stages based on distinct morphological features: the three stages (FI–FII–FIII) used in our classification were defined as follows: FI = small spindle shaped cell, FII = small epithelioid cells, FIII = large epithelioid cells. The L:E differentiation index was calculated analyzing the fibroblasts in early (E: FI + FII) and late (L: FII/FIII + FIII) differentiation stage (Herskind and Rodemann 2000). The quantitative analysis revealed that the percentages of cells of the FII and FIII groups, corresponding to more differentiated stages, was highly variable among the CHO-AFs (FII + FIII ranging from approximately 14 to 97 %), while more homogeneous percentages were found in the controls perilesional-Fs (ranging from approximately 58 to 78 %) and in MEAC-Fs (40 %) or NAS-Fs (61 %). Consistent with these percentages, also the L:E ratio was highly variable in CHO-AFs, as reported in Table 1 (representative examples are shown in Fig. 1b).

To evaluate the growth rate of the fibroblast cultures, we analyzed the cell cycle distribution by cytofluorimetry as described in “Materials and methods”. The results showed that all the CHO-AFs cultures were characterized by a lower percentage of cells in G2/M phase compared to control cultures (Table 1 and representative examples in Fig. 1c) and that a reduction of the mitotic ability was frequently related to a higher L:E ratio (Table 1).

Since it is well established that the activation of quiescent fibroblasts leads to the acquisition of a more differentiated phenotype and to an increased secretion of extracellular matrix components and proteases (for a recent review, see Räsänen and Vaheri 2010), we quantified by ELISA test, in cellular homogenates of CHO-AFs and control fibroblasts, the expression levels of the metalloproteases MMP-2 and MMP-9, which are known to be modulated in cholesteatoma tissues (Schönermark et al. 1996; Shibosawa et al. 2000). The concentrations of both MMP-2 and MMP-9 obtained from CHO-AFs were clearly higher compared to MEAC-Fs, NAS-Fs and perilesional-Fs homogenates (Fig. 1d; Table 1) and the CHO-AFs expressing increased amounts of MMPs correspond to less proliferating cultures (Table 1).

**Fig. 2:**
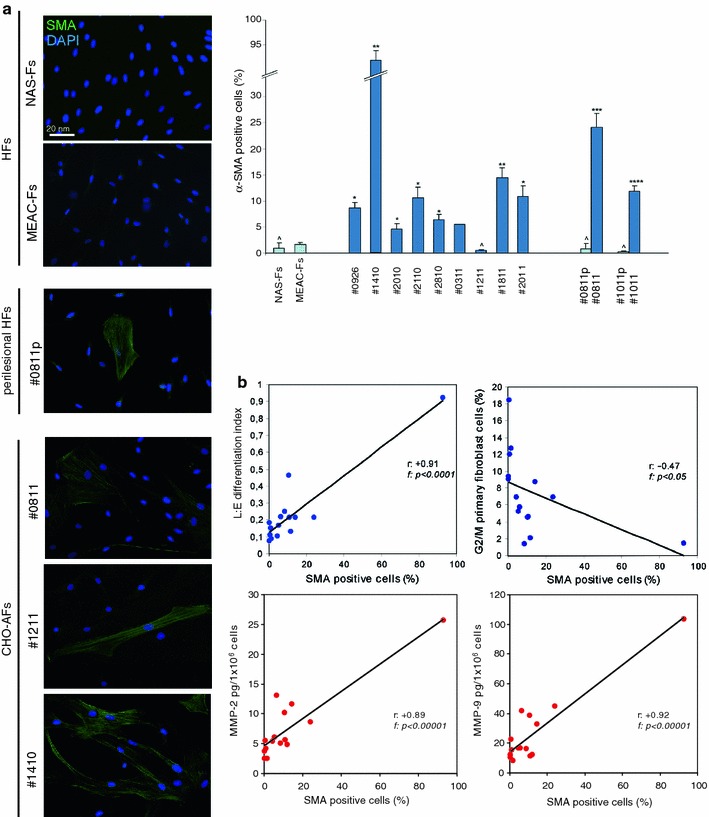
Evaluation of α-SMA expression. **a** Immunofluorescence analysis of the expression of the activation and differentiation markers anti α-SMA shows a signal localized in intracellular filaments, also organized in bundles, situated in the peripheral areas of the cytoplasm and enhanced in most of CHO-AFs compared to control cells. The percentage of α-SMA positive cells is higher in most CHO-AFs cultures respect to perilesional-Fs and control cells. Nuclei are stained with DAPI. *Bar* 20 μm. Student’s *t* test was performed and significance level has been defined as described in “Materials and methods”. Results reported in graph represent the mean values ± SE. Statistics: ^*p* = NS versus MEAC-Fs and *p* = NS versus MEAC-Fs; *p < 0.05 versus MEAC-Fs; **p < 0.01 versus MEAC-Fs; ***p < 0.001 versus MEAC-Fs and *p* < 0.001 versus corresponding perilesional-Fs, ****p < 0.01 versus MEAC-s and *p* < 0.01 versus corresponding perilesional-Fs. **b** Correlation by linear regression analysis between α-SMA positivity rates and differentiation parameters. The L:E ratio and the metalloproteinases-2 and -9 levels, show a strong positive correlation with α-SMA expression in the primary fibroblast cultures (*r*: +0.91, f: *p* < 0.0001; *r*: +0.89, f: *p* < 0.00001; *r*: +0.92, f: *p* < 0.00001); in contrast the fraction of cells in G2/M shows a negative correlation with α-SMA expression (*r*: −0.47, f: *p* < 0.05)

Because the expression of α-smooth muscle actin (α-SMA) is the most commonly used marker for activated and differentiated fibroblasts (Räsänen and Vaheri 2010), to better evaluate the differentiated phenotype of the CHO-AFs, we analyzed by quantitative immunofluorescence the percentage of cells positive for α-SMA: as shown in Fig. 2a, the immunofluorescence signal appeared compatible with actin bundles in the peripheral areas of the cytoplasm and was enhanced in most of CHO-AFs. In addition, the percentage of the α-SMA-positive cells was higher in the CHO cultures compared to perilesional-Fs and control cells (Fig. 2a). The percentages of α-SMA-positive cells were correlated with the parameters of activation/differentiation used above: the linear regression analysis showed a strong positive correlation between the expression rate of α-SMA and the L:E ratio and the MMP-2 and -9 levels in all the primary fibroblast cultures (*r*: +0.91, f: *p* < 0.0001; *r*: +0.89, f: *p* < 0.00001; *r*: +0.92, f: *p* < 0.00001), while a negative correlation was found for α-SMA with the G2/M data (*r*: −0.47, f: *p* < 0.05) (Fig. 2b). Taken together, these results indicate that, although showing a great variability in morphological shape and in biological behaviour, the CHO-AFs display a more differentiated and activated phenotype than the perilesional-Fs, NAS-Fs and MEAC-Fs. Interestingly, some of the CHO-AFs showing the differentiated/activated phenotype were isolated from cholesteatoma tissue samples characterized by an extended dermal inflammatory infiltrate (Table 1), assessed as previously described (d’Alessandro et al. 2010)*.*

### Supernatants from CHO-AFs enhance keratinocyte proliferation and differentiation through KGF/FGF7 release

**Fig. 3:**
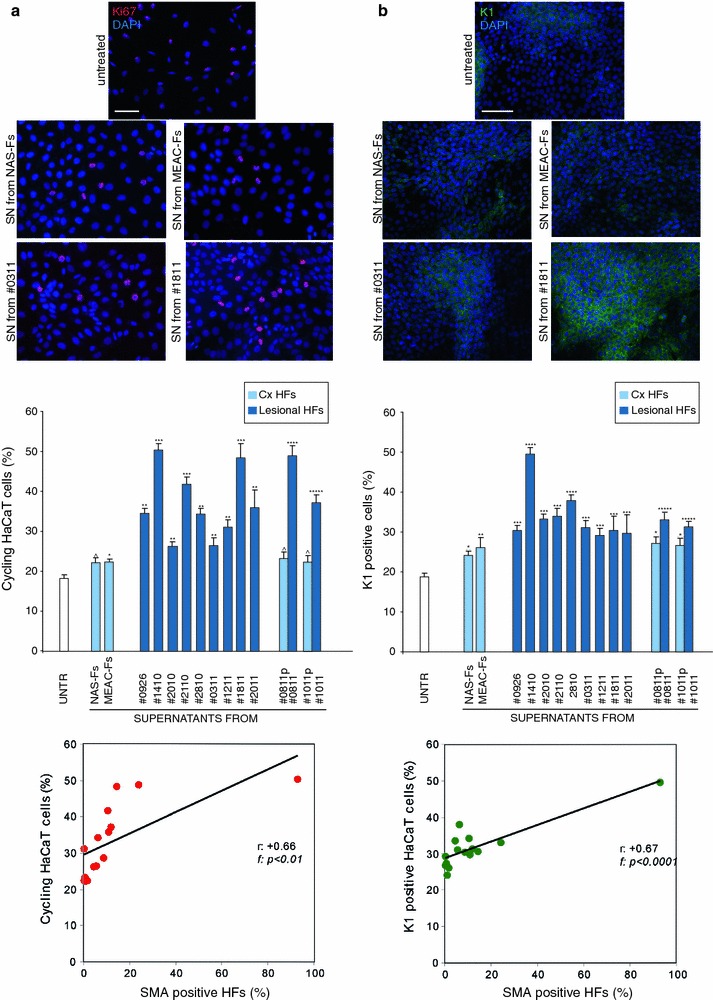
Biological effects induced by SNs from the fibroblast cultures on keratinocyte proliferation and differentiation. **a** HaCaT cells were serum-starved, treated with SNs of different primary cultures of fibroblasts for 48 h at 37 °C, fixed and immunostained with anti-Ki67 polyclonal antibodies, which identifies cycling cells. Quantitative immunofluorescence analysis indicates that the percentage of cells presenting positive nuclei was higher in HaCaT treated with SNs collected from CHO-AFs respect to that treated with SNs from control cells. The SNs from perilesional-Fs (#0811p and #1011p) induce a lower proliferating rate respect to SNs from the corresponding lesional tissue (#0811and #1011), but similar to SNs of control fibroblasts NAS-Fs and MEAC-Fs. At linear regression analysis, the proliferation rate induced by the different fibroblast derived SNs on HaCaT cells shows a positive correlation with α-SMA expression in the corresponding primary fibroblast cultures (*r*: +0.66; f: *p* < 0.01). Results reported in graph represent the mean values ± SE. Nuclei are stained with DAPI. *Bar* 20 μm. Student’s *t* test was performed and significance level has been defined as described in “Materials and methods”. Statistics: ^*p* < 0.05 versus untreated and *p* = NS versus MEAC-Fs; *p < 0.05 versus untreated; **p < 0.05 versus MEAC-Fs; ***p < 0.01 versus MEAC-Fs, ****p < 0.01 versus MEAC-Fs and *p* < 0.01 versus corresponding perilesional-Fs; *****p < 0.01 versus MEAC-Fs and *p* < 0.05 versus corresponding perilesional-Fs. **b** Confluent HaCaT cells were serum-starved, treated with SN as above, fixed and immunostained with anti-K1 polyclonal antibodies, which identify early differentiated cells. Quantitative immunofluorescence analysis reveals that SNs collected from CHO-AFs induce a higher differentiation respect to SNs from control cells. Cells treated with SNs from perilesional-Fs (#0811p and #1011p) appear less differentiated respect to those treated with SNs from the corresponding lesional tissue (#0811and #1011) and similar to SNs of NAS-Fs and MEAC-Fs. At the linear regression analysis, the percentage of K1 positive keratinocytes in response to the different SNs shows a positive correlation with α-SMA expression of the corresponding fibroblast (*r*: +0.67; f: *p* < 0.0001). Nuclei are stained with DAPI. *Bar* 50 μm. Results reported in graph represent the mean values ± SE. Student’s *t* test was performed and significance level has been defined as described in “Materials and methods”. Statistics: *p < 0.05 versus untreated and *p* = NS versus MEAC-Fs; **p < 0.05 versus untreated; ***p < 0.05 versus untreated and *p* = NS versus MEAC-Fs, ****p < 0.01 versus untreated and *p* < 0.01 versus MEAC-Fs; *****p < 0.05 versus MEAC-Fs and *p* < 0.05 versus corresponding perilesional-Fs

It is well known that there is a close interplay between the stromal cells and the epithelial counterpart; this interaction is mediated by soluble paracrine signals and secreted extracellular matrix and proteases released from mesenchymal cells which regulate the epithelial proliferation, differentiation and migration (Räsänen and Vaheri 2010). In order to analyze the possible biological activity of soluble factors produced by the cultured CHO-AFs on a cellular epithelial model, we treated the human keratinocyte HaCaT cell line with the culture supernatants (SNs) collected from CHO-AFs, NAS-Fs, MEAC-Fs and perilesional-Fs, to recreate in vitro the interaction that occurs in vivo between derma and epithelium. To first analyze the proliferative effect, HaCaT cells were serum starved, treated with SNs from CHO-AFs or from control cells for 48 h at 37 °C and then fixed and stained with antibodies against Ki67 antigen, which identify cycling cells (Fig. 3a). Quantitative immunofluorescence analysis indicated that the percentage of cells presenting positive nuclei was higher in keratinocytes treated with SNs collected from CHO-AFs than in those treated with SNs from NAS-Fs or MEAC-Fs. Moreover, the SNs from perilesional-Fs induced a lower proliferating rate than SNs collected from the corresponding lesional cultures, but similar to SNs of control fibroblasts (Fig. 3a). The growth rate induced by the different SNs was correlated with the percentage of α-SMA expression in the corresponding primary fibroblast cultures and the linear regression analysis revealed a significant positive correlation (*r*: +0.66; f: *p* < 0.01) (Fig. 3a).

To evaluate the ability of the SNs to induce epithelial differentiation, we analyzed the expression in keratinocytes of the early differentiation marker keratin 1 (K1). To this aim, HaCaT cells, which are known to undergo differentiation following cell confluence and stratification (Ryle et al. 1989; Capone et al., 2000), were let to grow up to confluence, serum starved and treated with SNs as above before fixation and staining with anti-K1 antibodies (Fig. 3b). Quantitative immunofluorescence analysis showed that SNs isolated from CHO-AFs induced a higher percentage of K1 positive HaCaT cells than the SNs from NAS-Fs and MEAC-Fs. Again, cells treated with SNs from perilesional-Fs appeared less differentiated than the cells treated with the SNs from the corresponding lesional cultures and similar to SNs from control cells (Fig. 3b). The percentage of K1 positive HaCaT cells induced by the different SNs was correlated with α-SMA expression as above and the linear regression analysis showed a significant positive correlation (*r*: +0.67; f: *p* < 0.0001) (Fig. 3b). Altogether, these results indicate that SNs from CHO-AFs have a greater ability to induce epithelial cell proliferation and differentiation in comparison with SNs from control fibroblasts.

**Fig. 4:**
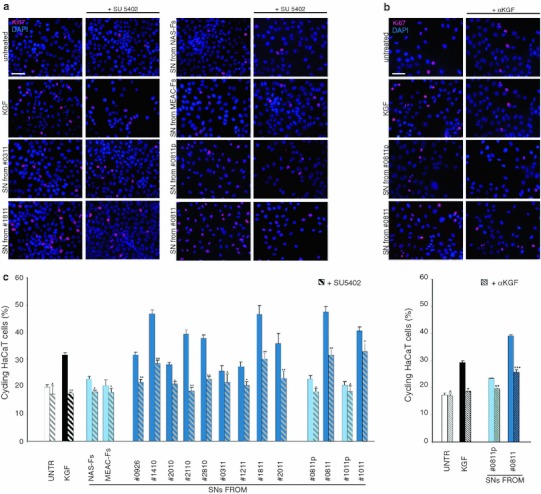
Reduction of the SN proliferation effects by inhibition of the KGFR activity or block of the KGF binding to the receptor. HaCaT cells were serum-starved, treated with SNs or with KGF for 48 h at 37 °C in presence of the specific FGFR tyrosine kinase inhibitor SU5402 (**a**) or of the neutralizing anti-KGF antibodies (**b**), fixed and immunostained with anti-Ki67 polyclonal antibodies. Quantitative immunofluorescence analysis indicates that inhibition of the KGFR activity as well as block of the KGF binding to the receptor induce a reduction in the percentage of cycling cells in response to the different SNs. The reduction is more evident after treatment with SNs from CHO-AFs, as well as after KGF treatment, respect to SNs from control cells. Nuclei are stained with DAPI. *Bar* 20 μm. **c** Results reported in graph represent the mean values ± SE. Student’s *t* test was performed and significance level has been defined as described in “Materials and methods”: Statistics: (left): ^*p* = NS versus corresponding untreated; *p < 0.05 versus corresponding untreated; **p < 0.01 versus corresponding untreated; (right): ^ *p* = NS versus corresponding untreated; *p < 0.01 versus corresponding untreated; **p < 0.05 versus corresponding untreated; ***p < 0.01 versus corresponding untreated

To verify whether the effects induced by SNs treatment on the HaCaT keratinocytes could be due to KGF released by the fibroblasts, the addition of the supernatants to the cells was performed in the presence of the specific FGFR tyrosine kinase inhibitor SU5402, similar to our previous in vitro study (Visco et al. 2009). Treatment with recombinant KGF 20 ng/ml in the presence or not of SU5402 was used as control. Cells were then fixed and stained with anti-Ki67 (Fig. 4a, c). The quantitative immunofluorescence analysis of cycling cells as above showed that, as expected (Belleudi et al. 2007), KGF treatment induced an increase in the percentage of Ki67 positive cells that was significantly reduced by the presence of the KGFR inhibitor. Similarly, SU5402 was able to inhibit the proliferative effects for all the SNs; however, the inhibition was much greater in the keratinocytes treated with the SNs isolated from CHO-AFs (Fig. 4a, c), suggesting that the proliferative activity of the SNs derived from CHO-AFs could be most attributable to an increased release of KGF in the culture medium. Comparable results were obtained by inhibiting the proliferative effect of the SNs through immunoblock of KGF with neutralizing anti-KGF antibodies (Fig. 4b, c), unequivocally indicating the relevance of the growth factor in the process.

**Fig. 5:**
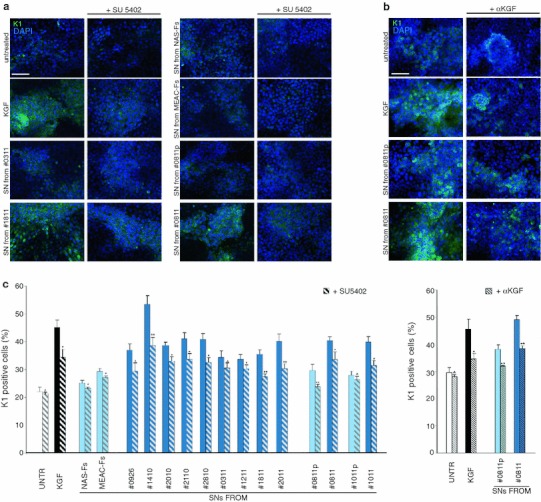
Reduction of the SN differentiation effects by inhibition of the KGFR activity or block of the KGF binding to the receptor. Confluent HaCaT cells were serum-starved, treated with SN or with KGF in presence of SU5402 (**a**) or anti-KGF antibodies (**b**) as above, fixed and immunostained with anti-K1 polyclonal antibodies. Quantitative immunofluorescence analysis shows that inhibition of KGFR activity as well as block of the KGF binding to the receptor reduce the differentiation marker expression induced by the different SNs. The reduction is more evident in HaCaT cells treated with SNs from CHO-AFs and is similar to that observed for KGF treatment. Nuclei are stained with DAPI. *Bar* 50 μm. **c** Results reported in graph represent the mean values ± SE. Student’s *t* test was performed and significance level has been defined as described in “Materials and method”. Statistics: (*left*): ^*p* = NS versus corresponding untreated cells; *p < 0.05 versus corresponding untreated cells; **p < 0.01 versus corresponding untreated cells; (*right*): ^ *p* = NS versus corresponding untreated cells; *p < 0.05 versus corresponding untreated cells; **p < 0.01 versus corresponding untreated cells

Since it is known that KGF promotes keratinocyte differentiation (Capone et al. 2000; Belleudi et al. 2011) and we have reported that in cholesteatoma tissue KGF up-modulation is associated with keratinocyte differentiation (d’Alessandro et al. 2010), to evaluate if the effects of the SNs on the expression of K1 could be ascribed to KGF, HaCaT cells were treated as above in the presence of SU5402, fixed and stained with anti-K1 antibodies (Fig. 5a, c). The quantitative analysis showed that the percentages of K1-positive cells in response to treatment either with the growth factor or the different SNs was reduced in the presence of the receptor inhibitor (Fig. 5a, c). The differentiative effects were more reduced by the inhibitor when the stimulation was obtained with the SNs collected from CHO-AFs (Fig. 5a, c) compared to the treatments with SNs from control fibroblasts, further indicating that KGF might represent the major paracrine effector secreted by the activated fibroblasts associated with the cholesteatoma lesions. Again, the immunoblock of KGF by the addition of neutralizing antibodies led to an inhibition of the differentiative effect totally comparable to that obtained through the use of SU5402 (Fig. 5b, c), confirming the key role played by KGF.

**Fig. 6:**
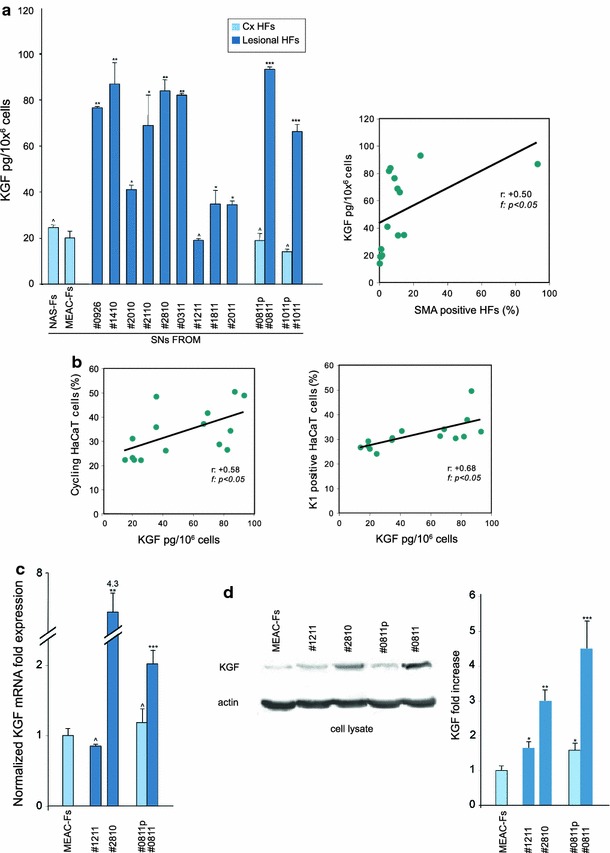
KGF expression and secretion in the culture SNs. **a** KGF-quantitation by ELISA test on SNs from the different cultures of fibroblasts. The KGF levels released in the CHO-AFs medium are variable but higher than in the SNs from control cells. The KGF amounts in different SNs show a positive correlation with α-SMA expression in the corresponding primary fibroblast cultures (*r*: +0.50; f: *p* < 0.05). Results reported in graph represent the mean values ± SE. Mann–Withney test was performed and significance level has been defined as described in “Materials and methods”. Statistics: ^*p* = NS versus MEAC-Fs; *p < 0.05 versus MEAC-Fs; **p < 0.01 versus MEAC-Fs; ***p < 0.01 versus MEAC-Fs and *p* < 0.01 versus corresponding perilesional-Fs. **b** In HaCaT cells treated with the different SNs, the proliferation rate and the percentage of K1 positive cells show a positive correlation with KGF levels released in the SNs (*r*: +0.58 and +0.68, respectively; *p* < 0.05). **c** KGF mRNA transcript levels were quantitated by real-time PCR: a clear fold increase in KGF mRNA expression is observed in lesional SNs compared to MEAC-Fs and perilesional-Fs. **d** Western blot analysis on the cell lysates shows that the specific band corresponding to KGF protein is increased in lesional Fs. The equal loading was assessed with anti-actin antibody. Statistics for the densitometric analysis: *p < 0.05 versus MEAC-Fs; **p < 0.01 versus MEAC-Fs; ***p < 0.01 versus MEAC-Fs and #0811p

In order to demonstrate efficient secretion of KGF in the medium from the fibroblast cultures, we performed Sandwich ELISA assay to analyze the KGF concentration in the culture supernatants. The results indicate that all fibroblast cell cultures were able to produce KGF in various amounts (Table 1; Fig. 6a). The concentrations of KGF obtained from CHO-AFs were clearly higher compared to perilesional-Fs, MEAC-Fs and NAS-Fs SNs (Fig. 6a). Interestingly, among the CHO-AFs, the #1410 whose SN contained high amounts of KGF and showed the greater ability to induce epithelial cell proliferation and differentiation, corresponded to activated and differentiated fibroblasts characterized by the highest percentage of SMA-positive cells and the greatest MMP levels. In contrast, the #1211 SN, which contained lower amounts of KGF, was collected from fibroblasts with a quiescent phenotype like control cells.

The relationship between the differentiation and activation degree of the fibroblast cultures and their ability to secrete KGF was demonstrated by linear regression analysis (Fig. 6a) that showed a positive correlation between the α-SMA expression rate of the cultures and the KGF levels in different SNs (*r*: +0.50; f: *p* < 0.05). In addition, the role of KGF in the HaCaT cell stimulation induced by SNs treatment was assessed by regression analysis (Fig. 6b): in fact, the KGF levels in the SNs showed a positive correlation with HaCaT proliferation (*r*: +0.58; f: *p* < 0.05) and differentiation rates (*r*: +0.68; f: *p* < 0.05).

To better define the molecular mechanisms leading to the increased KGF release in the culture supernatants from CHO-AFs and to ascertain if the variable amounts of KGF secreted in the medium would correspond to enhanced expression of the growth factor at both transcriptional and protein levels, we performed both RT-PCR and Western blot analysis on a selection of cultured fibroblasts characterized by highly different amounts of KGF secretion: the results shown in Fig. 6c and d demonstrated, compared to MEAC-Fs and perilesional-Fs, a clear increased mRNA and protein expression in those CHO-AFs #2810 and #0811, which displayed higher concentrations of the growth factor detected in ELISA test (Fig. 6c, d). In accordance with the conclusion that the enhanced KGF secretion corresponds to a real transcriptional up-regulation, very low levels of mRNA and protein expression were observed in CHO-AFs #1211 characterized by a quiescent phenotype and by a low amount of the growth factor released in the medium (Fig. 6c, d).

**Fig. 7:**
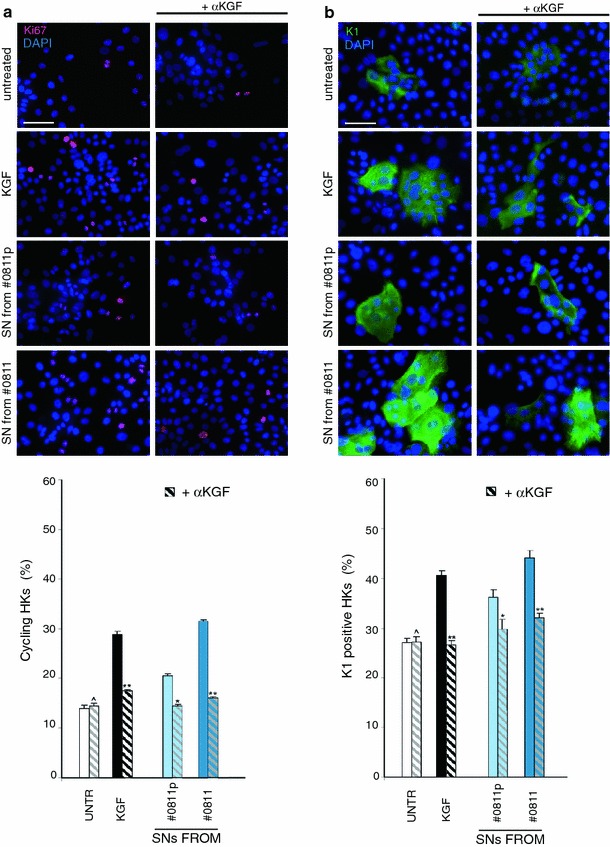
Proliferative and differentiative effects induced by SNs from the fibroblast cultures on primary cultured human keratinocytes. HKs were serum-starved, treated with SNs for 48 h at 37 °C, fixed and immunostained with anti-Ki67 (**a**) or with anti-K1 polyclonal antibodies (**b**), also in the in presence of netralizing anti-KGF antibodies as above. Quantitative immunofluorescence analysis (**c**) indicates that the percentage of Ki67-positive or K1-positive cells was higher in HKs treated with SNs collected from CHO-AFs in respect to that treated with SNs from control cells and that the block of the KGF binding to the receptor by addition of anti-KGF antibodies reduces the effects of SNs. Nuclei are stained with DAPI. *Bar* 20 μm. Statistics: (left): ^*p* = NS versus corresponding untreated cells; *p < 0.05 versus corresponding untreated cells; **p < 0.01 versus corresponding untreated cells; (right): ^*p* = NS versus corresponding untreated cells; *p < 0.05 versus corresponding untreated cells; **p < 0.01 versus corresponding untreated cells

To further demonstrate that the proliferative and differentiative effects induced by the SNs collected from the activated lesional fibroblasts represent a physiological/pathological phenomenon occurring in middle ear epidermis, we performed a parallel set of experiments using human primary cultured keratinocytes (HKs). As observed for HaCaT cells, the SN from lesional fibroblasts, but not that from the perilesional cells, was able to increase either the proliferation or the differentiation of the HKs (Fig. 7a, b). The addition of anti-KGF neutralizing antibodies during the incubation with the SNs as above caused a reduction of the percentage of cycling and K1-positive cells to the untreated levels (Fig. 7a, b), confirming the key role played by KGF in the keratinocyte stimulation.

**Fig. 8:**
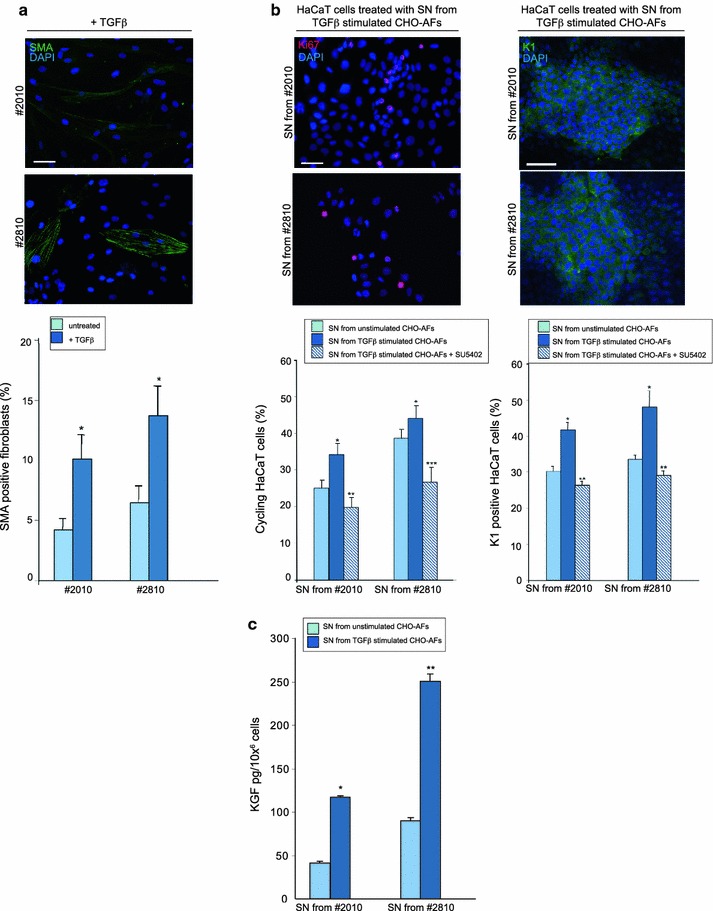
Evaluation of biological effects induced on HaCaT cells by SNs from CHO-AFs stimulated with TGFβ. **a** Two selected cultures of CHO-AFs, #2010 and #2810, were treated with TGFβ for 48 h at 37 °C and then fixed and stained with anti-α-SMA. Quantitative immunofluorescence analysis of the percentage of α-SMA positive cells shows that, respect to untreated cells, TGFβ treatment induces an increase of the α-SMA positive cells in both cultures. Nuclei are stained with DAPI *Bar* 20 μm. Results reported in graph represent the mean values ± SE. Student’s *t* test was performed and significance level has been defined as described in “Materials and methods”: Statistics: *p < 0.05 versus untreated. **b** HaCaT cells were serum-starved, treated for 48 h at 37 °C with the SN from #2010 and #2810 cultures treated with TGFβ as above. The treatments were performed also in the presence of the KGFR inhibitor SU5402. The cells were then fixed and immunostained with anti-Ki67 or with anti-K1 polyclonal antibodies. Quantitative immunofluorescence analysis of the Ki67 or K1 positive cells shows that the SNs from cultures treated with TGFβ induce an enhance the proliferation and differentiation of HaCaT cells respect to the SNs from untreated cultures. Inhibition of the KGFR activity reduces the effects of SNs. Nuclei are stained with DAPI. *Bars* 20 μm (left panel) and 50 μm (right panel). Results reported in graph represent the mean values ± SE. Student’s *t* test was performed and significance level has been defined as described in materials and methods. Statistics for proliferation analysis: *p < 0.05 versus SNs from unstimulated CHO-AFs, **p < 0.05 versus SNs from unstimulated #2010 and *p* < 0.01 versus SNs from TGFβ stimulated #2010, ***p < 0.05 versus SNs from unstimulated and TGFβ stimulated #2810. Statistics for differentiation analysis: * p < 0.01 versus SNs from unstimulated CHO-AFs, **p < 0.05 versus SNs from unstimulated CHO-AFs and *p* < 0.01 versus SNs from TGFb stimulated CHO-AFs. **c** KGF-quantitation by ELISA test on SNs from #2010 and #2810 cultures treated with TGFβ as above. In both cultures the amount of KGF released in the medium is higher in SNs from TGFβ treated cells respect to the SNs from untreated cells. Results reported in graph represent the mean values ± SE. Mann–Withney test was performed and significance level has been defined as described in “Materials and methods”. Statistics: *p < 0.05 and **p < 0.01 versus untreated

To prove that the secretion of the KGF from the primary cultures is related to the fibroblast differentiation degree, we used TGFβ treatment to induce in vitro differentiation of the CHO-AFs cultures. In fact, it is well documented that TGFβ promotes the differentiation and activation of quiescent stromal fibroblasts (Serini and Gabbiani 1999; De Wever et al. 2008). We selected two CHO-AFs moderately differentiated, #2010 and #2810, and we treated them with TGFβ 50 ng/ml for 48 h before fixation and immunostaining with anti α-SMA antibody as above. The quantitative immunofluorescence analysis showed that TGFβ treatment induced an increase in the number of α-SMA positive cells in both cultures (Fig. 8a). To verify if the most differentiated phenotype induced by TGFβ treatment could correspond to a greater capacity of the fibroblasts to stimulate the epithelial cells, HaCaT keratinocytes were serum starved and incubated with the SNs collected from the TGFβ treated cultures before fixation and staining with anti-Ki67 or with anti-K1 antibodies. The quantitative analysis revealed that SNs obtained from CHO-AFs stimulated with TGFβ had a greater capacity to induce HaCaT cell proliferation and differentiation than SNs isolated from unstimulated CHO-AFs (Fig. 8b). Again, this induction was reduced in the presence of the KGFR inhibitor SU5402, suggesting that the effects on the keratinocytes would be mostly mediated by the paracrine release of KGF (Fig. 8b). In agreement with this hypothesis, sandwich ELISA assay performed on these SNs demonstrated that the enhancement in proliferation and differentiation observed with the SNs from stimulated CHO-AFs is probably mediated by an increased release of KGF in the medium (Fig. 8c).

Altogether, these results suggest that the CHO-AFs are able to sustain the cholesteatoma growth and altered differentiation through the secretion of paracrine diffusible factors and that, among them, KGF might represent one of the major effectors.

## Discussion

In the last few years, it has been widely recognized that the dermal fibroblasts in an inflammatory microenvironment play a crucial role in the deregulation of the keratinocyte growth leading to epidermal hyperproliferation. Due to the difficulties in studying these complex epithelial-mesenchymal interactions in vivo, many efforts have been devoted to develop in vitro models for the analysis of the molecular mechanisms and autocrine or paracrine loops involved. The cholesteatoma of the middle ear, being characterized by keratinocyte hyperproliferation and chronic inflammation, represents a model pathology to study the pathogenesis of epithelial non-neoplastic diseases, in which activation of the stromal cells sustains the hyperproliferation and metaplastic altered differentiation. Similarly to the experimental studies conducted for other pathological conditions, different in vitro models have been developed also for the cholesteatoma disease: however, most of them aimed to culture and analyze cholesteatoma-derived keratinocytes, which appear to retain in vitro the altered behaviour observed in vivo (Cheshire et al. 1995; Albers-op t’ Hof et al. 2002; Hilton et al. 2011). The only 3D co-culture model, developed using both primary keratinocytes and fibroblasts from the cholesteatoma tissue, was useful to investigate the cytokeratin protein profile and metaplastic behaviour of the lesional epidermal cells and to ascertain the importance of the fibroblasts in the optimization of the in vitro system (Raynov et al. 2008); however, the study was not attempted to address the potential activation of the stromal components. Therefore, to investigate if also the cholesteatoma-associated fibroblasts might possess biological characteristics, which distinguish them form the normal quiescent fibroblasts and which could be retained at early passages in culture, in this study, we focused our attention to these cells, analyzing their secretory behaviour and their degree of differentiation or activation. We found that, compared to the perilesional or control normal fibroblasts, all cultures derived from cholesteatoma samples were less proliferating and more differentiated; moreover, although very variable among the cultures, the differentiated phenotype well correlated with an activated state, shown by MMP-2 and MMP-9 secretion, demonstrating that also in a non-neoplastic context, such as the cholesteatoma disease, the associated fibroblasts might exert a pathological role through biological functions which are maintained in culture.

**Fig. 9:**
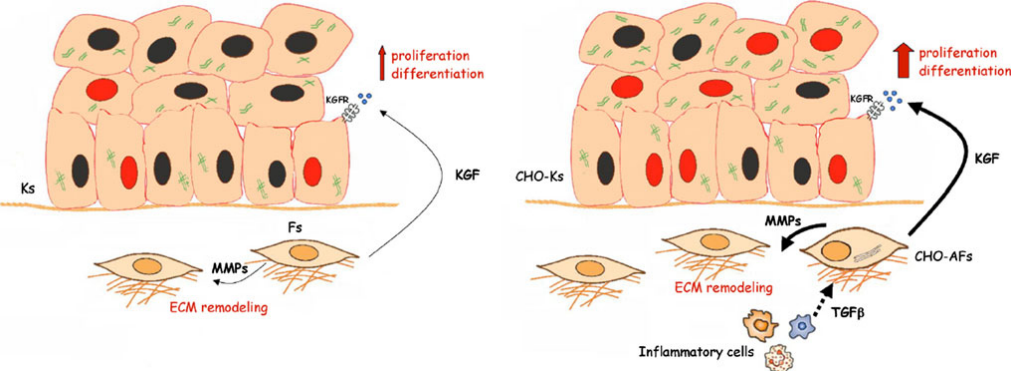
Schematic drawing of the proposed role of the cholesteatoma-associated fibroblasts (CHO-AFs) in the deregulation of epidermal proliferation and differentiation through a paracrine loop involving secretion of KGF: in the pathological framework (*right panel*), the CHO-AFs, activated by growth factors and cytokines (such as TGFβ) released by inflammatory cells, produce an increased amount of KGF compared to the physiological conditions (*left panel*). Through binding to its receptor KGFR, the growth factor increases keratinocyte proliferation and differentiation. The activated CHO-AFs release in the stroma also enhanced levels of MMPs which contribute in the remodeling and degradation of the extracellular matrix

Among the growth factors and cytokines which may play a role in the development of the cholesteatoma disease, as well as in other hyperproliferative epithelial pathologies, KGF appears a key molecular effector (Kojima et al. 1996; Ishibashi et al. 1997; Yamamoto-Fukuda et al. 2003, 2010; Barbara et al. 2008; d’Alessandro et al. 2010) and a possible target or biomarker for therapeutic intervention (Yamamoto-Fukuda et al. 2008). Many studies have reported that the expression of KGF is up-modulated in cholesteatoma, but none of them was aimed to analyze in detail the contribution of the KGF-secreting fibroblasts to the disease. Here, through the evaluation of the capacity of the culture supernatants to modulate the biological response in human keratinocytes in the presence of the KGFR inhibitor, we demonstrated the single key contribution of the KGF released in the medium in deregulating the proliferative and differentiative response. Moreover, the variable amount of KGF detected in the different supernatants well correlated with the quiescent or differentiated/activated phenotype of the cultured fibroblasts; this correlation is further demonstrated by the increase in KGF production observed following the widely used triggering of differentiation by TGFβ treatment. Therefore, as depicted in the schematic drawing in Fig. 9, the activation of the stromal fibroblasts present in the pathological tissue and the consequent increased secretion of KGF appear to play a crucial role in the deregulation of the epidermal proliferation and differentiation which characterizes the cholesteatoma tissue. Interestingly, we observed that the CHO-AFs showing the differentiated/activated phenotype were isolated from cholesteatoma tissue samples characterized by an extended dermal inflammatory infiltrate, assessed as described in our previous study (d’Alessandro et al. 2010), suggesting that the variability of the phenotypic changes in the different fibroblast populations would reflect the level of tissue inflammation. Therefore, we propose that this model might be very useful to evaluate the unknown pathogenetic role of the stroma perimatrix component of the cholesteatoma lesion and might contribute in general for a better understanding of the epithelial-mesenchymal interactions. Moreover, it is possible to assume that this model could be utilized for the in vitro assessment of the efficacy of therapeutic strategies directed to interfere with the pathological paracrine loops.
